# Lack of 24/7 Attending Physician Coverage in US Emergency Departments, 2022

**DOI:** 10.1016/j.acepjo.2025.100050

**Published:** 2025-02-05

**Authors:** Carlos A. Camargo, Krislyn M. Boggs, Ashley F. Sullivan, Janice A. Espinola, Maeve Swanton, Deborah D. Fletcher

**Affiliations:** 1 Department of Emergency Medicine, Massachusetts General Hospital, Boston, Massachusetts, USA; 2 Department of Emergency Medicine, Willis-Knighton Medical Center, Shreveport, Louisiana, USA

**Keywords:** attending physician, emergency department, rural, critical access hospital, physician coverage

## Abstract

**Objectives:**

The growth of nonphysician emergency department (ED) practitioners and the rural shortage of emergency physicians have raised concerns about the declining presence of physicians in EDs. Our objective was to identify the percentage of US EDs without 24/7 attending physician coverage and to investigate the location and characteristics of these EDs.

**Methods:**

The National ED Inventory (NEDI)-USA survey is sent annually to the ED director of every nonfederal US ED. The 2022 survey (administered in 2023 to all EDs open during 2022) included the question: “Is at least one attending physician (not resident) on duty in the ED 24 h/d?” The NEDI-USA database includes basic ED characteristics such as annual visit volume, critical access hospital (CAH) status, rural location, and freestanding ED status. We investigated the association of ED characteristics with a lack of 24/7 attending physician coverage.

**Results:**

The 2022 NEDI-USA database identified 5622 EDs, of which 4621 (82%) responded to the 24/7 attending physician question. Overall, 344 of 4621 (7.4%) EDs reported the absence of 24/7 attending physician coverage. In several states, ≥30% of the state EDs lacked 24/7 coverage; the states with the highest percentages were North Dakota (58%), South Dakota (56%), and Montana (46%). Among these 344 EDs, 318 (92%) had annual visit volumes <10,000. Most EDs (307 [89%] of 344) were in a CAH; 248 (72%) were rural, and 6 (2%) were freestanding.

**Conclusion:**

Approximately 1 in 13 US EDs lacks 24/7 attending physician coverage. The absence of 24/7 attending physician coverage was more common in low-volume EDs and CAHs. These observations highlight important gaps in ED care nationally. Changes in CAH regulations may help address this important workforce issue.

**The Bottom Line**

We investigated US emergency departments (EDs) without 24/7 attending physician coverage. Based on a national survey of ED directors, 1 in 13 EDs did not have at least 1 attending physician on duty 24/7. In several states, ≥30% of the EDs lacked 24/7 coverage. Among these 344 EDs, 92% had low annual visit volumes, 89% were in critical access hospitals, and 72% were rural. These observations highlight important gaps in ED care nationally. Changes in critical access hospital regulations may help address this workforce issue.

## 1 Introduction

### 1.1 Background and Importance

An essential characteristic of US emergency departments (EDs) is their availability 24 h/d, 7 d/wk (24/7) for anyone who seeks medical care.^1^ An American College of Emergency Physicians poll from 2021 suggests that nearly 80% of the US public report that they most trust a physician to lead their medical care while in the ED compared with a nurse, physician assistant, or nurse practitioner.^2^ This same American College of Emergency Physicians poll suggests that adults do make assumptions about whether their treating ED clinician is a physician (eg, a portion assume this based on the color of the clinician’s scrubs or whether they are wearing a stethoscope). However, the dramatic growth of nonphysician practitioners over the past 20 years^3^ and the rural shortage of emergency physicians^4^ have raised concerns about the declining presence of physicians in US EDs.

### 1.2 Goals of This Investigation

The objective of the current study was to identify the percentage of US EDs without 24/7 attending physician coverage and to investigate the location and characteristics of these EDs.

## 2 Methods

### 2.1 Study Design

We performed a cross-sectional study of US EDs. The Mass General Brigham Human Research Committee reviewed this study and classified it as exempt.

### 2.2 National ED Inventory-USA

The National ED Inventory (NEDI)-USA survey is sent annually to the ED director of every nonfederal, nonspecialty hospital US ED. Federal EDs (eg, those affiliated with Veterans Administration, military, or Indian Health Service) are excluded because they are not truly “open” to the general public. Specialty hospital EDs (eg, the ED of a psychiatric hospital) are not generally capable of managing the broad spectrum of injury and disease that is cared for in the vast majority of US EDs.

### 2.3 Outcomes: 24/7 Attending Physician Coverage

The NEDI-USA survey is sent first by email or mail up to 3 times, and then nonresponding ED directors are contacted by telephone to complete the survey by interview.^5^^,^^6^ We identified EDs with the absence of 24/7 attending physician coverage through the survey question: “Is at least one attending physician (not resident) on duty in the ED 24 h/d?” (yes/no) If no, ED directors were asked: “When a physician is not on duty in the ED, is any physician available to the ED by 2-way voice communication 24/7 – from within your hospital (yes/no/not applicable [eg, freestanding ED]) or from outside your hospital (yes/no/not applicable [eg, freestanding ED]).”

The NEDI-USA database includes basic ED characteristics such as annual visit volume, critical access hospital (CAH) status, rural location (based on presence outside of a core-based statistical area),^7^ hospital-based vs freestanding ED status, and receipt of telehealth services. EDs are further characterized by special capabilities, such as having a burn center,^8^ trauma center,^9^ stroke center,^10^ pediatric emergency care coordinator (PECC),^11^ or geriatric ED recognition.^12^ Adult trauma centers and stroke center status are further characterized as “basic” or “advanced” centers. “Basic” adult trauma centers have certification equivalent to that of the American College of Surgeons' level III verification, and “Advanced” adult trauma centers have certification equivalent to that of American College of Surgeons' level I or II verification.^9^ “Basic” stroke centers have certification equivalent to that of The Joint Commission’s Acute Stroke Ready Hospital, and “Advanced” stroke centers have certification equivalent to that of The Joint Commission’s Primary Stroke Center, Thrombectomy-Capable Stroke Center, or Comprehensive Stroke Center certification.^10^

### 2.4 Data Analyses

Data analysis included descriptive statistics. Specifically, we determined the proportion and characteristics of EDs without 24/7 attending physician coverage. Because of the potential of confounding, we also fit a logistic regression model to determine independent predictors of lack of 24/7 attending physician coverage. All analyses were completed using Excel (Microsoft) and Stata 15 (StataCorp). To examine the national distribution of lack of 24/7 attending physician coverage, a choropleth map was created using ArcGIS (Esri).

## 3 Results

The 2022 NEDI-USA database identified 5622 EDs, of which 4621 (82%) responded to the 24/7 attending physician question. Overall, 4277 (92.6%) responded “yes,” whereas 344 (7.4%) responded “no” (ie, these EDs reported that they did not have 24/7 attending physician coverage). Although 7.4% of all US EDs reported that they lacked 24/7 attending physician coverage, their geographic distribution revealed large between-state differences (Fig). In 15 states, zero responding EDs reported that they lacked 24/7 attending physician coverage. Two states that have recently introduced legislation requiring 24/7 in-person physician coverage in EDs, Indiana and Virginia,^13^^,^^14^ had no EDs that reported a lack of 24/7 coverage. However, in several states (shown in white), ≥30% of the state’s EDs lacked 24/7 coverage. The states with the highest percentages were North Dakota (58%), South Dakota (56%), and Montana (46%).

Among these 344 EDs without 24/7 attending physician coverage, the annual visit volumes were <10,000 for 318 (92%), whereas 23 (7%) had 10,000 to 19,999 and 3 (<1%) had ≥20,000. Most EDs (307 [89%] of 344) were in a CAH; 248 (72%) were rural, and 6 (2%) were freestanding. Overall, 266 (77%) of the 344 EDs without 24/7 coverage received telehealth, and 78 (23%) did not. Compared with EDs with 24/7 attending physicians, EDs without 24/7 physicians were more likely to have annual visit volumes <10,000, to be in the Midwest and rural areas, to be a CAH, and to receive telehealth services. They were less likely to be freestanding, to be adult trauma centers, to be stroke centers, and to have PECCs (Table 1).

**Table 1:** Presence of 24/7 attending physicians in US emergency departments, n = 4621.

ED characteristic	EDs with 24/7 attending physician (n = 4277; 92.6%)	EDs without 24/7 attending physician (n = 344; 7.4%)	*P* value
	n (%)	n (%)	
Annual visit volume < 10,000			<.001
Yes	1073 (25)	318 (92)	
No	3204 (75)	26 (8)	
Region			<.001
Northeast	501 (12)	17 (5)	
Midwest	1065 (25)	198 (58)	
South	1904 (45)	66 (19)	
West	807 (19)	63 (18)	
Critical access hospital			<.001
Yes	898 (21)	307 (89)	
No	3379 (79)	37 (11)	
Rural location			<.001
Yes	712 (17)	248 (72)	
No	3565 (83)	96 (28)	
Freestanding ED			<.001
Hospital-based ED	3623 (85)	338 (98)	
Freestanding ED	654 (15)	6 (2)	
Adult trauma center			<.001
Yes	860 (20)	2 (0.6)	
No	3417 (80)	342 (99)	
Stroke center			<.001
Yes	2054 (48)	95 (28)	
No	2223 (52)	249 (72)	
Pediatric emergency care coordinator			<.001
Yes	1036 (24)	41 (12)	
No	3241 (76)	303 (88)	
Receives telehealth services			.03
Yes	3056 (72)	266 (77)	
No	1201 (28)	78 (23)	

ED, emergency department.

Regarding the special capabilities of these 344 EDs, there were no adult or pediatric burn centers. Two EDs were adult trauma centers (both basic); none were pediatric trauma centers. Ninety-five (28%) were stroke centers, with 89 basic and 6 advanced. Only 41 (12%) of the 344 EDs reported having a PECC. None were recognized as a geriatric ED.

In multivariable analysis, independent predictors of the absence of 24/7 attending physician coverage included annual visit volume <10,000 and CAHs. Rural location, hospital-based (vs freestanding) ED status, receipt of telehealth services, and lack of adult trauma center certification were also associated with a lack of 24/7 coverage (Table 2).

**Table 2:** Associations between emergency department characteristics and lack of 24/7 attending physician coverage; n = 4621 US emergency departments.

ED characteristic	Unadjusted OR (95% CI)^a^	Adjusted OR (95% CI)^b^
Annual visit volume < 10,000	36.52 (24.34-54.8)	9.84 (6.17-15.69)
Critical access hospital	31.22 (22.02-44.26)	4.67 (3.01-7.24)
Rural location	12.93 (10.08-16.59)	1.79 (1.33-2.41)
Freestanding ED	0.10 (0.04-0.22)	0.38 (0.15-0.93)
Receives telehealth services	1.34 (1.03-1.74)	1.36 (1.01-1.83)
Adult trauma center	0.02 (0.01-0.09)	0.16 (0.04-0.66)
Stroke center	0.41 (0.32-0.53)	1.07 (0.80-1.43)
Pediatric emergency care coordinator	0.42 (0.3-0.59)	0.90 (0.61-1.31)

OR > 1 indicates higher odds of lack of 24/7 attending physician coverage.

ED, emergency department; OR, odds ratio.

a Association between individual factor and outcome (n = 344 cases); 8 models.

b One model with all factors liste.

Among the 344 EDs without 24/7 attending physician coverage, 50% did not have 2-way voice communication 24/7 with any physician within the hospital, and 19% did not have this with any physician outside the hospital. Ten EDs (3%) reported “no” to both subquestions (ie, they lacked 24/7 2-way voice communication with any physician).

## 4 Limitations

The study limitations include self-reported data from ED leadership and an 82% national response rate. We speculate that many ED directors are reluctant to report a lack of 24/7 attending physician coverage and, for similar reasons, that nonresponding EDs are more likely to lack this coverage. That said, compared with responding EDs, nonresponding EDs were more often freestanding adult trauma centers and stroke centers. They were less often CAHs and less often in rural areas (Table S1). Some of these characteristics suggest that nonresponding EDs are more likely to have 24/7 attending physicians, and others suggest that they are less likely.

## 5 Discussion

Based on a national survey of ED directors, we found that at least 7.4% of US EDs lack 24/7 attending physician coverage (approximately 1 in 13 EDs). This percentage varied widely by state. EDs that were rural and were in CAHs were more likely to lack 24/7 attending physician coverage.

The geographic distribution of the 344 EDs lacking 24/7 coverage revealed a disproportionate number in the central US Indeed, the Figure closely resembles our prior work on the emergency physician workforce, where we reported lower numbers of emergency physicians in this same geographic area.^3^ This prior work also found a lack of emergency physicians in rural areas, which was again consistent with the lack of 24/7 physician coverage in rural EDs, as reported in the current study. As reported previously, there has been a rise in nonphysician practitioners over the past 20 years, and they are supplementing the lack of physicians in rural EDs.^3^^,^^4^ This remains potentially concerning, given differences in training and care patterns between physician and nonphysician practitioners.^15^^,^^16^

**Figure:**
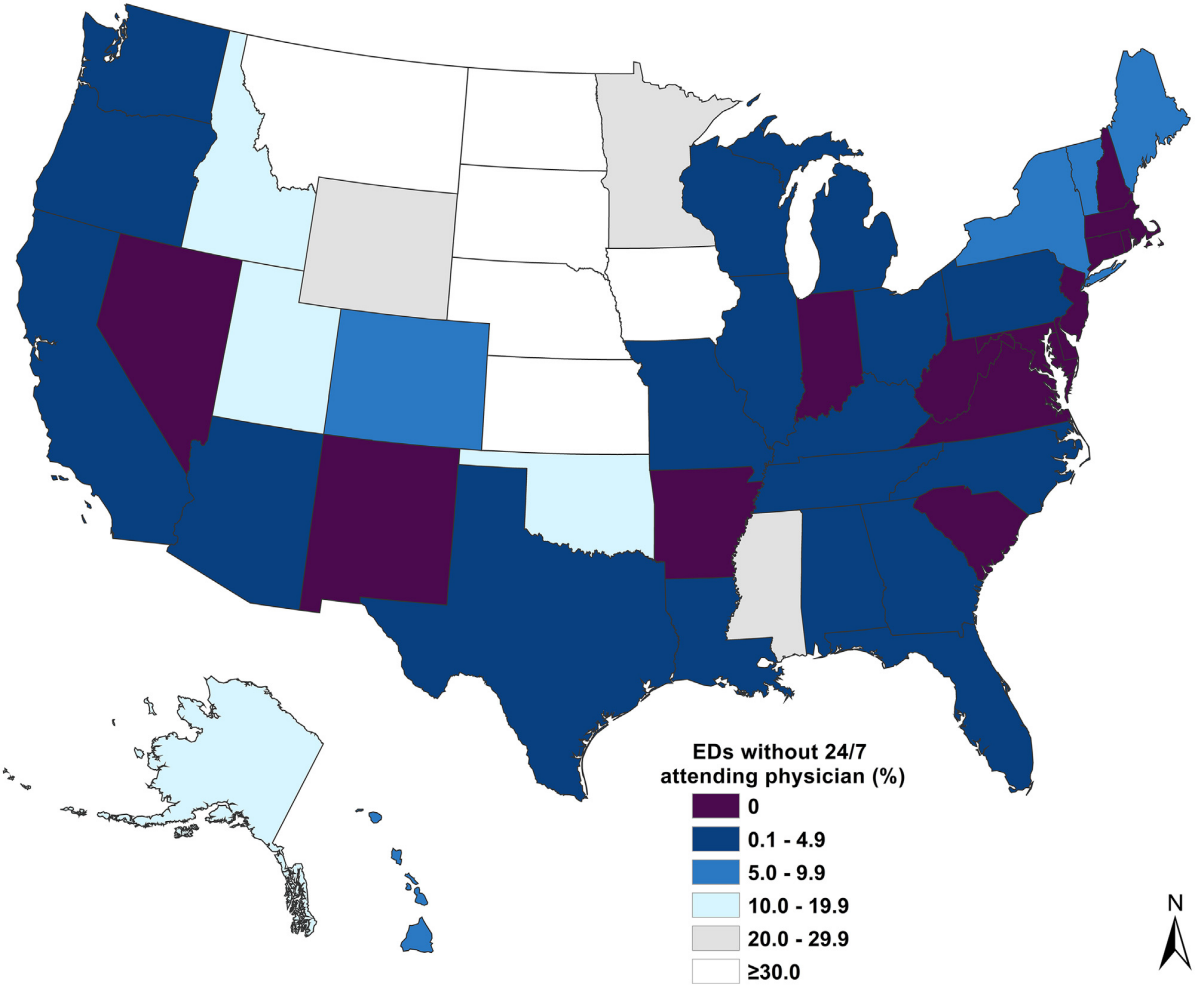
Percentage of US emergency departments without 24/7 attending physician coverage by state, 2022.

The characteristics of EDs without 24/7 coverage are not surprising: they are low-visit volume EDs (<10,000 visits/y) located within a CAH and a rural area. Although 77% of these EDs receive telehealth, 23% do not. Furthermore, a small percentage (3%) of these EDs reported a lack of 2-way voice connection 24/7 with any physician, either within or outside of the hospital. In a prior survey of rural EDs, we found that most ED directors without telehealth reported that their ED, hospital, or health system leadership had considered it, but the start-up and maintenance costs were often cited for the lack of telehealth adoption. The current results remind us of this persistent, untapped opportunity for quality improvement.^17^

Most (89%) EDs without 24/7 attending physician coverage were in a CAH. Briefly, CAH designation is given to eligible rural hospitals by the Centers for Medicare and Medicaid Services. The goal of the CAH program is to reduce the financial vulnerability of these designated hospitals and to improve access to health care by keeping these hospitals open. By definition, CAHs provide access to health care in areas that otherwise would have a dearth of local hospital-based care. Although CAH regulations require that each facility operates a 24/7 ED, they do not require that the ED be staffed by an attending physician 24/7.^18^ A revision in CAH policy, including financial support for 24/7 attending physician coverage, may help to reduce the number of EDs operating without this coverage.

In summary, approximately 1 in 13 US EDs lack 24/7 attending physician coverage, which is more common in low-volume EDs and CAHs. These observations highlight critical gaps in emergency care in the US Changes in CAH regulations, along with increased public awareness, may help address this important workforce issue.

## Author Contributions

C.A.C. and D.D.F. conceived the study, and C.A.C., K.M.B., A.F.S., J.A.E., and M.S. designed the study. C.A.C., K.M.B., and A.F.S. supervised the study. K.M.B. and M.S. obtained the data, and K.M.B., J.A.E., and M.S. managed the data. J.A.E. provided statistical advice and analyzed the data. C.A.C. drafted the manuscript, and all authors contributed substantially to its revision. C.A.C. takes responsibility for the paper as a whole.

## Funding and Support

By *JACEP Open* policy, all authors are required to disclose any and all commercial, financial, and other relationships in any way related to the subject of this article as per ICMJE conflict of interest guidelines (see www.icmje.org). The authors have stated that no such relationships exist.

## Conflict of Interest

All authors have affirmed they have no conflicts of interest to declare.
