# Supplementary material for: Lack of 24/7 Attending Physician Coverage in US Emergency Departments, 2022
Source: J Am Coll Emerg Physicians Open. 2025 Feb 5;6(2):100050. doi: 10.1016/j.acepjo.2025.100050 (PMC11847254; doi:10.1016/j.acepjo.2025.100050)
Supplement: Supplementary Table 1 [file mmc1.docx]

**Table S1**. 2022 National Emergency Department Inventory-USA Survey: Responders vs Non-responders, N=5,622

|  | **Responders (n=4,657, 83%)** | **Non-Responders (n=965, 17%)** |  |
| --- | --- | --- | --- |
| **ED Characteristic** | **n (%)** | **n (%)** | **P-value** |
| Annual visit volume <10,000 |  |  | 0.06 |
| Yes | 1,399 (30) | 261 (27) |  |
| No | 3,258 (70) | 704 (73) |  |
| Region |  |  | 0.34 |
| Northeast | 522 (11) | 108 (11) |  |
| Midwest | 1,276 (27) | 244 (25) |  |
| South | 1,987 (43) | 441 (46) |  |
| West | 872 (19) | 172 (18) |  |
| Critical Access Hospital |  |  | <0.001 |
| Yes | 1,209 (26) | 148 (15) |  |
| No | 3,448 (74) | 817 (85) |  |
| Rural location |  |  | <0.001 |
| Yes | 962 (21) | 127 (13) |  |
| No | 3,695 (79) | 838 (87) |  |
| Freestanding ED |  |  | 0.005 |
| Hospital-based ED | 3,989 (86) | 792 (82) |  |
| Freestanding ED | 668 (14) | 173 (18) |  |
| Adult Trauma Center |  |  | 0.03 |
| Yes | 869 (19) | 209 (22) |  |
| No | 3,788 (81) | 756 (78) |  |
| Stroke Center |  |  | 0.02 |
| Yes | 2,166 (47) | 489 (51) |  |
| No | 2,491 (53) | 476 (49) |  |
| Geriatric ED |  |  | 0.32 |
| Yes | 275 (6) | 49 (5) |  |
| No | 4,382 (94) | 916 (95) |  |
| Adult Burn Center |  |  | 0.83 |
| Yes | 67 (1) | 13 (1) |  |
| No | 4,590 (99) | 952 (99) |  |

Abbreviation: ED, emergency department
